# Motor Imagery Classification via Kernel-Based Domain Adaptation on an SPD Manifold

**DOI:** 10.3390/brainsci12050659

**Published:** 2022-05-18

**Authors:** Qin Jiang, Yi Zhang, Kai Zheng

**Affiliations:** 1 College of Computer Science and Technology, Chongqing University of Posts and Telecommunications, Chongqing 400065, China; namy_jiang@hotmail.com; 2 School of Advanced Manufacturing Engineering, Chongqing University of Posts and Telecommunications, Chongqing 400065, China; zhengkai2001@163.com; 3 Advanced Manufacturing and Automatization Engineering Laboratory, Chongqing University of Posts and Telecommunications, Chongqing 400065, China

**Keywords:** EEG, brain–computer interfaces, domain adaptation, subspace learning, symmetric positive definite matrices, Riemannian manifolds

## Abstract

Background: Recording the calibration data of a brain–computer interface is a laborious process and is an unpleasant experience for the subjects. Domain adaptation is an effective technology to remedy the shortage of target data by leveraging rich labeled data from the sources. However, most prior methods have needed to extract the features of the EEG signal first, which triggers another challenge in BCI classification, due to small sample sets or a lack of labels for the target. Methods: In this paper, we propose a novel domain adaptation framework, referred to as kernel-based Riemannian manifold domain adaptation (KMDA). KMDA circumvents the tedious feature extraction process by analyzing the covariance matrices of electroencephalogram (EEG) signals. Covariance matrices define a symmetric positive definite space (SPD) that can be described by Riemannian metrics. In KMDA, the covariance matrices are aligned in the Riemannian manifold, and then are mapped to a high dimensional space by a log-Euclidean metric Gaussian kernel, where subspace learning is performed by minimizing the conditional distribution distance between the sources and the target while preserving the target discriminative information. We also present an approach to convert the EEG trials into 2D frames (E-frames) to further lower the dimension of covariance descriptors. Results: Experiments on three EEG datasets demonstrated that KMDA outperforms several state-of-the-art domain adaptation methods in classification accuracy, with an average Kappa of 0.56 for BCI competition IV dataset IIa, 0.75 for BCI competition IV dataset IIIa, and an average accuracy of 81.56% for BCI competition III dataset IVa. Additionally, the overall accuracy was further improved by 5.28% with the E-frames. KMDA showed potential in addressing subject dependence and shortening the calibration time of motor imagery-based brain–computer interfaces.

## 1. Introduction

A brain–computer interface (BCI) provides a direct control pathway between the human brain and external devices, without relying on peripheral nerve and muscle systems [1]. BCIs have demonstrated potential in medical rehabilitation, education, smart homes, and so on. Most non-invasive BCIs are based on EEG signals, and the neural response patterns are decoded by well-designed algorithms, which can convert movement intentions into computer commands to control external devices, such as a wheelchair [2], an artificial limb [3], a spelling system [4,5], or a quadcopter [6]. Steady-state visual evoked potential (SSVEP), P300, and motor imagery (MI) are widely studied neural response paradigms for BCIs. SSVEP and P300 have shown breakthroughs in spelling applications [4,5,6], while MI is prized for its simple stimulus paradigm design, and allows subjects to express motor intention in a natural way [7].

Motor imagery is accompanied by event-related desynchronization/event-related synchronization (ERD/ERS) in the functional motor area [8,9], and effective characterization of the ERD/ERS phenomenon is paramount for a motor imagery-based brain–computer interface (MI-BCI) system. Due to non-stationary EEG signals, the conventional BCI system requires users to undergo a long period of training to obtain substantial labeled instances for a robust model. However, long and monotonous training not only causes a psychological burden to users, but also jeopardizes the adaptability of the BCI system [10,11]. Domain adaptation has been developed to deal with limited training data from the target by employing data from other sources. The objective of domain adaptation is to transfer useful knowledge from a source group into the target training set, to overcome the problem of limited calibration data [12]. As a result, a well-performing classifier can be obtained without a large number of labeled EEG samples from the target subject, thus shortening the training time.

However, the large inter-subject variability of EEG signals has been an impediment to domain adaptation learning. Borgwardt et al. [13] proposed a maximum mean discrepancy (MMD) criterion for comparing cross-domain distributions. MMD is nonparametric and can estimate the distance between the means of two domains without requiring any labels. Based on MMD, many effective domain adaptation methods have been derived. Transfer component analysis (TCA) learns a common subspace across domains in a Reproducing Kernel Hilbert Space (RKHS) to minimize the distance between the sample means of the source and target data [13]. The joint distribution adaptation (JDA) method measures the distributional discrepancy using the unweighted sum of marginal and conditional MMDs [14], and the balanced distribution adaptation (BDA) method leverages the importance of the marginal and conditional distribution [15]. Domain transfer multiple kernel learning (DTMKL) learns a linear combination of multiple kernels by minimizing both the distribution mismatch between the source and target domains, and the structural risk [16]. Manifold embedded distribution alignment learns a domain-invariant classier in the Grassmann manifold with structural risk minimization, while performing dynamic distribution alignment by considering the differing importance of marginal and conditional distributions [17].

Although the abovementioned domain adaptation methods have performed well in computer vision and image sets’ classification, they cannot be directly applied to the EEG signal classification. Since each column (row) of an EEG record is a non-stationary time series signal, it requires a relatively stable descriptor, such as the mean, variance, entropy, or power spectrum. The description of the MI-EEG characteristics has a great impact on the domain adaptation. The common spatial pattern (CSP) is a widespread feature extractor. In order to improve CSP’s ability to handle the interference of noise and non-stationarities in the EEG signals, many improved CSPs have been proposed. Regularization CSP (RCSP) improves the generalization ability of CSP by adding a priori information into the estimation of the inter-class covariance matrix [18,19,20]. The filter bank-based CSP subdivides EEG signals into several sub-bands to find the most discriminative features [21,22]. A temporally constrained sparse group spatial pattern (TSGSP) [23] jointly extracted the significant features from both the filter bank and multiple time windows. In [24], Dempster–Shafer theory (DST) was employed for the feature selection rules for internal selection. Additionally, combining CSP with domain adaptation approaches provides an effective means for feature extraction in a cross-subject scenario. The complex common spatial pattern (CCSP) linearly combines the inter-class covariance matrices according to the Kullback–Leibler (KL) divergence between the target and sources [25]. The sparse group representation model (SGRM) constructs a composite dictionary matrix with CSP features from both the target and other subjects [11]. However, CSP is a supervised feature extractor, and CSP will fail when the sample set is small or there is no label in the target domain. The covariance matrix descriptor of the EEG signal provides a way to circumvent feature extraction. 

Barachant et al. pioneered the use of the geometric structure of the EEG signal covariance matrix, and proposed the minimum distance to the Riemannian mean algorithm (MDRM) [26] and linear classification algorithms in tangent space (TSVM) [27]. The results outperformed complex and highly parametrized CSP classifiers. Additionally, covariance matrices as features yielded competitive results on the classification of evoked potentials, such as SSVEP [28], and event-related potentials, such as P300 [29]. By capitalizing on the geometric properties of the symmetric positive definite (SPD) matrices, many domain alignment techniques have been proposed to make the time-series data from different sessions/subjects comparable. The parallel transport (PT) [30] projected the SPD matrices from different subsets to a common tangent space, and the Riemannian Procrustes analysis [31] aligned the statistical distributions of two datasets using simple geometric transformations, and after the alignment in the Riemannian manifold, all cross-subject covariance matrices were mapped into a shared tangent space to train a classifier. The manifold embedded transfer learning (METL) [32] aligned the covariance matrices of the EEG trials on the SPD manifold, and then learned a domain-invariant classifier of the tangent vectors’ features by combining the structural risk minimization of the source domain and joint distribution alignment of source and target domains. Similarly, the manifold embedded knowledge transfer (MEKT) framework [33] first whitened the SPD matrices of cross-subjects to an identity matrix, and then performed domain adaptation using tangent vectors to minimize the joint probability distribution shift between the source and the target domains, while preserving their geometric structures. 

Assuming that the number of recording electrodes is C, then the dimension of the corresponding tangent vector is C(C+1)/2. With the increase of C, the dimension of the vector will expand rapidly, and may even exceed the number of training samples, resulting in over-fitting of the classifier [34,35]. In addition, the reference point has a great influence on the tangent plane, and the tangent space determined by different reference points varies greatly [36].

The SPD matrices have been proven to be a powerful data representation approach for images or image sets via covariance [37], region covariance descriptors [38], or sparse coding of the covariance matrices [39,40]. In MI-BCI, the second-order statistics of the EEG signal contain discernible information about the subject’s mental state, and the most widespread problem-solving idea is to decompose the covariance matrix and extract the projection vectors with large inter-class variance. CSP is a typical algorithm derived from this concept. Moreover, many studies have demonstrated that the Riemannian metric is more effective than Euclidean distance in describing the discrepancy between covariance matrices [37,38,39,40,41]. For the non-linearity of the Riemannian manifold, three approaches have been summarized in the literature: (i) Given that a manifold is a topological space with local Euclidean properties, the Riemannian manifold is locally flattened via tangent spaces. (ii) Under the assumption that the intrinsic structure of the data is inherently low-dimensional, several dimensionality reduction algorithms have been designed to discover the intrinsic low-dimensional manifold, such as Locally Linear Embedding, Isometric Feature Mapping, and Locality Preserving Projection. (iii) One could embed the manifold in a high-dimensional Reproducing Kernel Hilbert Space (RKHS), where subspace learning can be carried out. This concept has been confirmed in image set classification [38,42], but it has not yet been applied to motor imagery classification.

In light of the above, we proposed a kernel-based Riemannian manifold domain adaptation (KMDA) method to sidestep the tedious process of feature extraction and take advantage of Riemannian geometry, while avoiding the dimensional explosion of tangent vectors. In our framework, we considered the covariance matrices of EEG signals as features, and aligned the covariance matrices of the source and target in the Riemannian manifold. Then, the log-Euclidean-based Gaussian kernel permitted us to embed the manifold in a high-dimensional RKHS, wherein subspace learning was performed by minimizing the conditional distribution distance between the sources and the target, while preserving the discriminative information of the target. Additionally, we present a feature construction scheme for converting the EEG timing series into 2D frames, which not only fully exploits the electrode position and frequency band of the signal, but also further reduces the dimension of the covariance matrix.

To sum up, the main contributions of this paper include:

(1) The KMDA classifies the motor imagery tasks without any target labels.
(2) The KMDA defines a subspace learning framework in a RKHS space defined by a kernel on the SPD manifold.
(3) The KMDA not only minimizes the marginal and conditional distributions, but also considers intra/inter-class discriminative information of sources and the principal components of the target.
(4) A feature construction scheme is presented to reduce the dimension of the SPD matrix and the computational cost.

The rest of the paper proceeds as follows: Section 2 introduces the Riemannian metric theory of the SPD manifold, and the definition of Gaussian kernel applicable for Riemannian manifolds. Section 3 details our proposed framework, Section 4 provides a detailed description of the experiment design and results on three datasets, Section 5 presents a series of discussions, and a conclusion is drawn in Section 6. 

## 2. Preliminaries

This section provides an overview on the geometry of the symmetric positive definite (SPD) manifold and some Riemannian metrics for the kernel method. $Sym_{d}^{+}$ denotes the space spanned by the d×d SPD matrices, and $T_{p}$ is the tangent space on the point of $P\in Sym_{d}^{+}$. $X_{i}\in R^{c\times t}$ represents the single trail of recorded EEG signal with c electrodes and t time samples. $C_{i}$ represents a covariance matrix in Euclidean space, and $P_{i}$ is the point in the Riemannian manifold. ${\left\|X\right\|}_{F}=\sqrt{Tr(X^{T}X)}$ designates the Frobenius norm, ${\left(.\right)}^{T}$ denotes the transpose operator, and Tr(.) is the sum of the diagonal elements. The principal matrix exponential, $\exp(.):Sym_{d}^{+}\to Sym_{d}$, is defined as $\exp(X)=Udiag(\exp(\lambda_{1},\lambda_{2},\ldots ,\lambda_{n}))U^{T}$; similarly, the matrix logarithmic operator $\log(.):Sym_{d}\to Sym_{d}^{+}$ is defined as $\log(X)=Udiag(\log(\lambda_{1},\lambda_{2},\ldots ,\lambda_{n}))U^{T}$, with $X=Udiag(\lambda_{1},\lambda_{2},\ldots ,\lambda_{n})U^{T}$. $Exp_{P}(.)$ and $Log_{P}(.)$ denote the exponential and logarithmic maps at the reference point P, respectively.

### 2.1. Riemannian Metrics

The covariance matrix of a single trial, $X_{i}$, was normalized with the total variance, as follows:

(1) $$P_{i}=\frac{X_{i}\cdot {\left(X_{i}\right)}^{T}}{Tr(X_{i}\cdot {\left(X_{i}\right)}^{T})}$$

A covariance matrix is a typical symmetric positive definite (SPD) matrix, $P_{i}\in Sym_{d}^{+}$, and the value of its determinant is a direct measure of the dispersion of the associated multivariate Gaussian [43]. However, Euclidean geometry forms a non-complete space [35], which often leads to a swelling effect in regression or average operations [44] that for the determinant of the Euclidean mean can be strictly larger than the original determinants [35,43], giving spurious variation to the data. To fully circumvent these issues, Riemannian metrics are proposed for the SPD manifold.

Tangent Space: The covariance matrices of multi-channel EEG signals define an SPD space, which is locally homeomorphic to the Euclidean space, i.e., the topological manifold is a locally differential manifold [43,45]. The curvatures of the curves that pass through each point on the smooth differential manifold define a linear approximation space, also known as the tangent space. For the SPD manifold, there exists a pair of mappings transporting points from the manifold to its corresponding tangent space, and vice versa. Specifically, the logarithmic map is used to embed the neighbors of a given point into the tangent space with the point as a reference, and the exponential map reverses a tangent vector back to the manifold:

(2) $$\left\{\begin{array}{l} Sym_{d}^{+}\to T_{P}:S_{i}=Log_{P}(P_{i})=P^{\frac{1}{2}}\log(P^{-\frac{1}{2}}P_{i}P^{-\frac{1}{2}})P^{\frac{1}{2}}\\ T_{P}\to Sym_{d}^{+}:P_{i}=Exp_{P}(S_{i})=P^{\frac{1}{2}}\exp(P^{-\frac{1}{2}}S_{i}P^{-\frac{1}{2}})P^{\frac{1}{2}} \end{array}\right.$$

**Figure 1:**
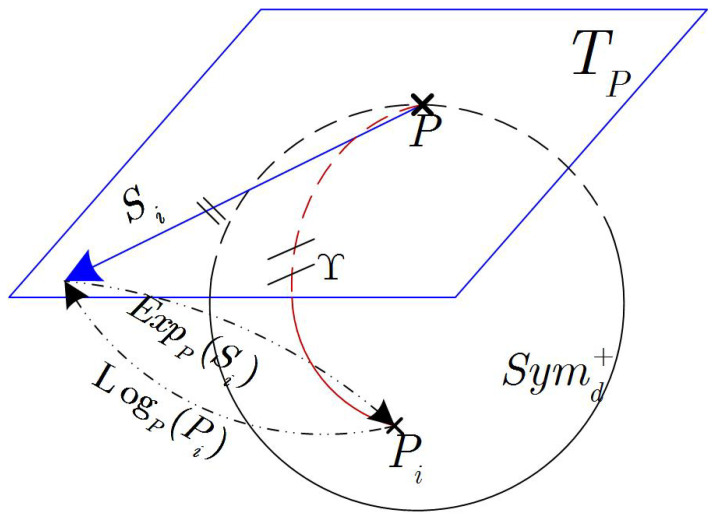
The mapping between the SPD manifold and the tangent plane at the point P.

As depicted in Figure 1, any vector in $T_{P}$ is identified as a geodesic starting at point P on the manifold; conversely, any bipoint $\left(P,P_{i}\right)$ can be mapped into a vector of $T_{P}$. It is worth noting that the tangent space of a manifold is not unique and depends on the reference point. Conventionally, the reference point is either an identity matrix or the Riemannian mean.

Riemannian mean: The Riemannian mean is defined as the point minimizing the following metric dispersion:

(3) $$M_{R}=\underset{P^{*}\in S_{+}^{d}}{\arg\min}\sum_{i=1}^{N}\delta_{R}{}^{2}(P^{*},P_{i})$$

where $\delta_{R}(P^{*},P_{i})$ denotes a distance suitable for the SPD manifold. Formula (3) does not define a closed-form solution [41], but it can be solved through an iterative algorithm [30]. 

Affine-Invariant Riemannian Metric: The affine-invariant Riemannian metric (AIRM) is a powerful and pervasive metric endowed to the SPD manifold, with the properties of uniquely defining the geodesic between two metrices and the mean of a set of metrices.

An arbitrary invariant distance on $Sym_{d}^{+}$ satisfies $\delta_{R}(P_{i},P_{j})=\delta_{R}(AP_{i}A^{T},AP_{j}A^{T})$, where A is a real invertible d×d matrix. Choosing $A=P_{i}{}^{-1/2}$, this distance is transformed to be a distance to the identity: $\delta_{R}(P_{i},P_{j})=\delta_{R}(I_{n},P_{i}{}^{-1/2}P_{j}P_{i}{}^{-1/2})$, where the affine-invariant Riemannian distance between two points, $P_{i}$ and $P_{j}$, is transformed to be the Riemannian distance between $P_{i}{}^{-1/2}P_{j}P_{i}{}^{-1/2}$ and $I_{n}$. Based on this, the distance $\delta_{R}(P_{i},P_{j})$ can be solved by calculating the geodesic distance of $P_{i}{}^{-1/2}P_{j}P_{i}{}^{-1/2}$ starting at the identity matrix, which amounts to calculating the vector of $P_{i}{}^{-1/2}P_{j}P_{i}{}^{-1/2}$ in the tangent space of $I_{n}$. Hence, the Affine-Invariant Riemannian Metric (AIRM) distance, $\delta_{air}$, between $P_{i}$ and $P_{j}$ is defined as:

(4) $$\delta_{air}(P_{i},P_{j})={\left\|\log(P_{i}{}^{-1/2}P_{j}P_{i}{}^{-1/2})\right\|}_{F}$$

Equivalently, we can write (4) as:

(5) $$\delta_{air}(P_{i},P_{j})={\left(\sum_{i=1}^{n}\log^{2}\lambda_{i}\right)}^{1/2}$$

where $\lambda_{i}$ is the eigenvalues of $P_{i}{}^{-1}P_{j}$.

Log-Euclidean Metric: The log-Euclidean metric (LEM) can also define the real geodesic distance of two SPD matrices [46], by computing the distance between their corresponding tangent vectors at the identity matrix, and we have:

(6) $$\delta_{lem}(P_{i},P_{j})={\left\|\log(P_{i})-\log(P_{j})\right\|}_{F}$$

Let $P=U\Sigma U^{T}$ be the eigen-decomposition of SPD matrix P, and Σ is the diagonal matrix of the eigenvalue. Its logarithmic map can be computed easily by: $\log(P)=U\log(\Sigma )U^{T}$. Compared with the AIRM, the log-Euclidean consumes less computation, while conserving excellent theoretical properties.

In addition to LEM and AIRM, another two metrics derived from Bergman divergences, namely Stein and Jeffrey divergence, are extensively used in manifold analysis. Stein and Jeffrey divergence are symmetric and affine invariants [41], which prompts the choice of these metrics in the Riemannian mean. Algorithm 1 illustrates the iterative process of estimating a Riemannian mean by AIRM.

**Algorithm 1.** Riemannian mean by AIRM.Input: Training set ${\left\{X_{i}\right\}}_{i=1}^{N}$, iteration Num, and termination criteria ε
Output: The Riemannian mean $M_{R}\in S_{+}^{n}$

Initialize the reference matrix C with an identity matrix.Calculate the covariance matrices of training samples $P_{i}\in S_{+}^{n}$ by (1)for *i* = 1: Num  Map each matrix $P_{i}$ to the tangent space at C by (2).  Obtain their Arithmetic mean $S_{i}$ in the tangent space.  Embed the Arithmetic mean $S_{i}$ to Riemannian space by (2), obtaining corresponding matrix $C_{i}$  if $\left|\right|C-C_{i}||<\varepsilon$
      break for;  end if  C←$C_{i}$end for$M_{R}$←C


### 2.2. Positive Definite Kernel on Manifold

Embedding into RKHS through kernel methods is a well-established and prevalent approach in machine learning [14]. However, embedding SPD manifolds into RKHS requires the kernel functions to be positive definite. The Gaussian kernel has worked well in mapping the data from Euclidean space into an infinite dimensional Hilbert space. In Euclidean, the Gaussian kernel is expressed as $\kappa (x_{i},x_{j}):=\exp(-{\left\|x_{i}-x_{j}\right\|}^{2}/2\delta^{2})$, which relies heavily on the Euclidean distance of two points. To define a Gaussian kernel applicable to the Riemannian manifold, a naive means is to replace the Euclidean distance with the geodesic distance on the premise that the generated kernel is positive definite.

Therefore, we defined the kernel: $\kappa :(Sym_{d}^{+}\times Sym_{d}^{+})\to \mathbb{R}$ by $\kappa (P_{i},P_{j}):=\exp(-\delta^{2}(P_{i},P_{j})/2\sigma^{2})$ for all points, $P_{1},\ldots ,P_{N}\in Sym_{d}^{+}$. κ is a positive definite kernel for all σ>0 only if the Riemannian geodesic metric, $\delta^{2}(P_{i},P_{j})$, is negative definite [42]. Herein, we consider the log-Euclidean metric as the geodesic distance, and we need to prove $\sum_{i,j}^{m}a_{i}a_{j}\delta^{2}(P_{i},P_{j})\leq 0$ for all m∈ℕ with $\sum_{i}^{m}c_{i}=0$. It is easy to prove that κ is a symmetric function: $\kappa (P_{i},P_{j})=\kappa (P_{j},P_{i})$, for all matrixes in the SPD manifold.

We analyzed the positive definiteness of the log-Euclidean metric as follows:

(7) $$\begin{array}{l} \sum_{i,j}a_{i}a_{j}\delta^{2}(P_{i},P_{j})=\sum_{i,j}a_{i}a_{j}||\log(P_{i})-\log(P_{j})|{|}_{F}^{2}\\ \Rightarrow \sum_{i,j}a_{i}a_{j}<\log(P_{i})-\log(P_{j}),\log(P_{i})-\log(P_{j})>\\ \Rightarrow \sum_{j}a_{j}\sum_{i}a_{i}<\log(P_{i}),\log(P_{i})>-2\sum_{i,j}a_{i}a_{j}<\log(P_{i}),\log(P_{j})>\\ +\sum_{i}a_{i}\sum_{j}a_{j}<\log(P_{i}),\log(P_{i})>\\ \Rightarrow -2\sum_{i,j}a_{i}a_{j}<\log(P_{i}),\log(P_{j})>\\ \Rightarrow -2||\sum_{i}a_{i}\log(P_{i})|{|}_{F}^{2}\leq 0 \end{array},$$

The Equation (7) provides the proof that the log-Euclidean kernel guarantees the positive definite of the Riemannian kernel, and satisfies the Mercer theorem. 

## 3. Proposed Framework

We assume that the sources have Ns labeled instances ${\left\{(X_{i}^{s},y_{i})\right\}}_{i=1}^{Ns}$, where $X_{i}^{s}\in R^{c\times t}$ denotes a single recorded EEG signal in the source domain, and $y_{i}\in \{1,\ldots ,l\}$ is the corresponding label. $\left\{X_{i}^{s}\right\}$ may be collected from one subject or from multiple subjects. ${\left\{X_{i}^{t}\right\}}_{1}^{Nt}$ is a collection of unlabeled records from the target. We assume that there is the same feature space and label space between domains, but, due to dataset shift, the marginal and conditional probability distribution are different. We use ϕ(x), $x\in Sym_{d}^{+}$ to map the feature vector to the RKHS space.

In this section, we elaborate on the proposed KMDA framework. KMDA aims to classify the unlabeled target data by exploiting the labeled data from multiple source domains. For the sake of simplicity, only one source domain is considered.

**Figure 2:**
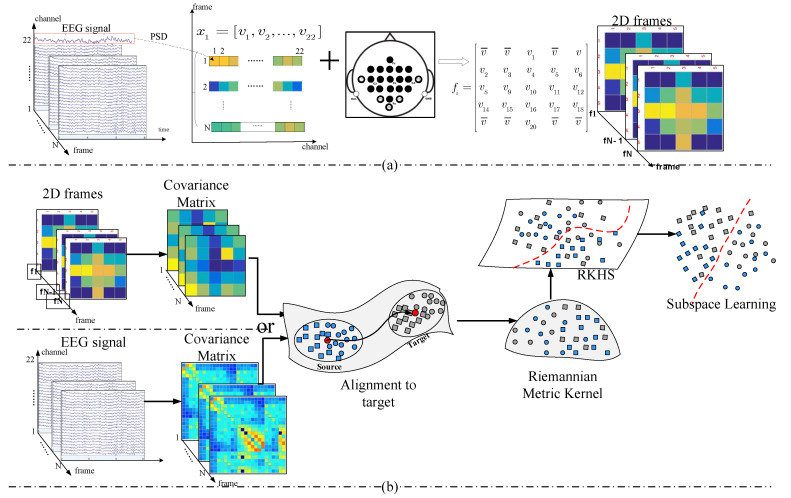
The illustration of proposed kernel-based Riemannian manifold domain adaptation. (**a**) The framework of converting the chain-like EEG signal into 2D frames. (**b**) The flow chart of KMDA with two types of covariance descriptor.

In KMDA, we take the covariance matrix of each EEG record as the feature. Covariance matrices define a symmetric positive definite space (SPD) that can be described by the Riemannian metrics. Due to individual differences in response patterns, and the deviation of the electrode installation position, there is a domain shift between the source and target covariance matrices. Hence, we first performed an alignment in the Riemannian manifold (RA). Subsequently, we embedded the manifold space into a high-dimensional Euclidean space through the log-Euclidean Gaussian kernel, where a discriminative subspace was learned. Alternatively, the SPD matrices can be defined by a set of 2D frames converted from a set of EEG records. Figure 2 shows the overall workflow of KMDA.

### 3.1. Alignment of the SPD Matrices

The correlation alignment (CORAL) has proven that aligning the second-order statistics can effectively mitigate the distribution differences across domains [47]. Referring to CORAL, we proposed an alignment in the Riemannian manifold, referred to as Riemannian alignment (RA), to align the symmetric positive definite matrices on the Riemannian manifold, which skillfully skips the tedious process of feature extraction from the EEG signal.

In RA, we whitened the source domain first to remove the correlations of the source domain, by:

(8) $$P_{i}^{s^{\prime }}={\left(\xi_{R}^{s}\right)}^{-1/2}P_{i}^{s}{\left(\xi_{R}^{s}\right)}^{-1/2}$$

Then, we recolored the source with correlations of the target domain.

(9) $$P_{i}^{st}={\left(\xi_{R}^{t}\right)}^{1/2}P_{i}^{s^{\prime }}{\left(\xi_{R}^{t}\right)}^{1/2}$$

where $P_{i}^{st}$ denotes the source matrix after recollection, and $\xi_{R}^{t}$ is the Riemannian mean of the target obtained through Algorithm 1. Equation (9) was used to reconstruct the source matrices using the target Riemannian mean, and after that, the source and target distributions differed little, so they can be considered to have an identical marginal probability distribution.

### 3.2. Kernel on Riemannian Manifold

Due to the non-Euclidean geometry of Riemannian manifolds, Euclidean algorithms yield inferior results on SPD matrices. We defined a Gaussian radial basis function-based positive definite kernel on the Riemannian manifold to embed the SPD manifold in a high-dimensional Reproducing Kernel Hilbert Space. The kernel makes it possible to utilize algorithms developed for linear spaces on nonlinear manifold data.

We employed the log-Euclidean distance as the Riemannian metric. One reason for this is that the log-Euclidean distance defines the real geodesic distance between two symmetric positive definite matrices, and more importantly, the Gaussian kernel with the log-Euclidean metric yields a positive definite kernel, satisfying the conditions of Mercer’s theorem, as proven by (7).

The SPD matrices can be transformed into the RKHS with:

(10) $$\kappa (P_{i},P_{j}):=\exp(-{\left\|\log(P_{i})-\log(P_{j})\right\|}_{F}^{2}/2\sigma^{2}),$$

### 3.3. Learning Mapping Matrix

Since the RA reconstructs the source using the eigenvectors and eigenvalues of the target, we assumed that the marginal distribution of the source and target remains identical in RKHS. The purpose of KMDA is to learn a transformation matrix, W, in RKHS, so as to minimize the conditional divergence of the source and target while maximizing the variance of the target domain and preserving the discriminative information of the source domain as much as possible.

(1) Target Variance

For an effective subspace, it should maximize the preservation of the principal components of the target and avoid projecting the features into irrelevant dimensions. Since the target labels are unknown, variance is used to measure the distinguishable information of target features. Hence, the objective function is defined as:

(11) $$\underset{W}{\max}Tr(W^{T}S_{t}W)$$

(2) Source Discriminative Information

The discriminative information of the source domain should be preserved in the new subspace. To this end, we exploited the labels to define the discernibility of the source; that is, we maximized the distance between classes while minimizing the distance within classes:

(12) $$\left\{\begin{array}{l} \underset{W}{\max}Tr(W^{T}S_{b}W)\\ \underset{W}{\min}Tr(W^{T}S_{w}^{\left(c\right)}W) \end{array}\right.$$

where $S_{w}^{\left(c\right)}$ is the within-class scatter matrix of the source data, $S_{b}=\sum_{c=1}^{l}Nt^{\left(c\right)}(m_{s}^{\left(c\right)}-{\bar{m}}_{s}){\left(m_{s}^{\left(c\right)}-{\bar{m}}_{s}\right)}^{T}$ is the between-class scatter matrix, in which $Nt^{\left(c\right)}$ is the number of source data of c-class, $m_{s}^{\left(c\right)}$ is the mean of samples from class c, and ${\bar{m}}_{s}$ is the mean of all source data.

(3) Condition Distribution

In the new subspace, the discrepancy between samples of the same type in the source and the target domain should be small, i.e., the conditional distribution distance should be minimized. We used the MMD as the criterion to measure the distribution divergencies.

(13) $$\begin{array}{l} dist(P(y_{t}|W^{T}\phi (P_{t})),P(y_{s}|W^{T}\phi (P_{s})))\\ ={\sum_{c=1}^{l}\left\|\frac{1}{N_{t}^{c}}\sum_{i=1}^{N_{t}^{c}}W^{T}\phi (P_{t})-\frac{1}{N_{s}^{c}}\sum_{i=1}^{N_{s}^{c}}W^{T}\phi (P_{s})\right\|}_{F}^{2} \end{array}$$

Then, we obtained the objective function:

(14) $$\underset{W}{\min}\sum_{c=1}^{l}Tr(W^{T}KLW)$$

where

K=KtKstKtsKs(L)ij=1(Nt(c))2,Pi,Pj∈Dt(c)1(Ns(c))2,Pi,Pj∈Ds(c)−1(Ns(c)·Nt(c)),Pi∈Ds(c),Pj∈Dt(c)Pi∈Dt(c),Pj∈Ds(c)0,otherwise

$K_{t}$$K_{s}$$K_{ts}$ are the kernel matrices defined by (10) on the Riemannian manifold in the target domain, source domain, and cross-domain, respectively.

(4) Overall Objective Function

Combining all of the above optimization objectives, we formulated the overall objective function of the proposed KMDA method:

(15) $$\underset{W}{\max}\frac{\alpha Tr(W^{T}S_{t}W)+\beta Tr(W^{T}S_{b}W)}{\mu \sum_{c=1}^{l}Tr(W^{T}\phi (P)M_{c}\phi^{T}(P)W)+\beta Tr(W^{T}S_{w}^{\left(c\right)}W)}\;\Rightarrow \underset{W}{\max}\frac{Tr(W^{T}[\begin{array}{cc} \alpha S_{t} & 0\\ 0 & \beta S_{b} \end{array}]W)}{\sum_{c=1}^{l}Tr(W^{T}[\begin{array}{cc} \mu K_{tt} & \mu K_{st}\\ \mu K_{ts} & \mu K_{ss}+\beta S_{w}^{\left(c\right)} \end{array}]LW)}$$

We simplified (15) as:

(16) $$\begin{array}{c} \underset{W}{\max}Tr(W^{T}[\begin{array}{cc} \alpha S_{t} & 0\\ 0 & \beta S_{b} \end{array}]W)\\ s.t.\;\;\sum_{c=1}^{l}Tr(W^{T}\left[\begin{array}{cc} \mu K_{t} & \mu K_{st}\\ \mu K_{ts} & \mu K_{s}+\beta S_{w}^{\left(c\right)} \end{array}\right]LW)=I \end{array}$$

where α, β, and μ are the trade-off parameters to balance the importance of each term.

By the Lagrange operator, we deformed the optimization function into:

(17) $$J=Tr(W^{T}[\begin{array}{cc} \alpha S_{t} & 0\\ 0 & \beta S_{b} \end{array}]W)+Tr(\lambda W^{T}(\sum_{c=1}^{l}[\begin{array}{cc} \mu K_{t} & \mu K_{st}\\ \mu K_{ts} & \mu K_{s}+\beta S_{w}^{\left(c\right)} \end{array}]L)W-I)$$

By setting $\frac{\partial J}{\partial W}=0$, we found:

(18) $$\left[\begin{array}{cc} \alpha S_{t} & 0\\ 0 & \beta S_{b} \end{array}\right]W=\lambda \sum_{c=1}^{l}\left[\begin{array}{cc} \mu K_{t} & \mu K_{st}\\ \mu K_{ts} & \mu K_{s}+\beta S_{w}^{\left(c\right)} \end{array}\right]LW$$

The optimal $W^{*}$ are given by the k leading eigenvectors of the eigen-decomposition of (18). 

Let $K_{t}=\phi {\left(P_{t}\right)}^{T}\phi (P_{t})$ and $K_{s}=\phi {\left(P_{s}\right)}^{T}\phi (P_{s})$, then we get $S_{t}=KH_{t}{\left(K_{t}\right)}^{T}$, $S_{w}^{\left(c\right)}=K_{s}^{\left(c\right)}H_{s}^{\left(c\right)}{\left(K_{t}^{\left(c\right)}\right)}^{T}$, $H_{s}^{\left(c\right)}=I_{Ns}-$$\frac{1}{Ns^{\left(c\right)}}1_{Ns}1_{Nt}{}^{T}$, and $H_{t}=I_{Nt}-\frac{1}{Nt}1_{Nt}1_{Nt}{}^{T}$, where $H_{t}$ is the center matrix, $I_{Nt}$ is the Nt×Nt identity matrix, and $1_{Nt}$ is the column vector with all ones. In $S_{b}$, we get $m_{s}^{\left(c\right)}=\frac{1}{Ns^{\left(c\right)}}\sum_{i=1}^{Ns^{\left(c\right)}}k_{i}^{\left(c\right)}$ and ${\bar{m}}_{s}=\frac{1}{Ns}\sum_{i=1}^{Ns}k_{i}$, with $k_{i}=\phi {\left(P_{s}\right)}^{T}\phi (P_{i})$, $P_{i}\in D_{s}$.

Given a new instance, $P_{t}\in Sym_{d}^{+}$, from the target, its projection, $z_{t}$, in the discriminant subspace was obtained by: $z_{t}=W^{*}K_{tt}$, where $K_{tt}=[k(P_{1},P_{t}),\ldots ,k(P_{N},P_{t})]$ and N=Nt+Ns. The classification was performed on the classifier trained with the source data. 

The pseudo-codes of the KMDA algorithm are described in Algorithm 2.

**Algorithm 2.** Kernel-Based Manifold Domain Adaptation.Input: EEG and source labels: ${\left\{(X_{i}^{s},y_{i})\right\}}_{i=1}^{Ns}$, ${\left\{X_{i}^{t}\right\}}_{1}^{Nt}$;   Parameters: α=1, μ=1, β, k.Output: Transformation matrix: $W^{*}$; Target labels $Y_{t}$.
1.Calculate covariance matrices ${\left\{P_{i}^{s}\right\}}_{i=1}^{Ns}$ and ${\left\{P_{i}^{t}\right\}}_{i=1}^{Nt}$.1′.  Or Calculate the covariance matrices of 2D frames.2.Calculate the Riemannian means of source and target by Algorithm 1.3.Align the SPD matrices of source and target by (8) and (9)4.Initialize pseudo labels of target domain $Y_{t}^{\prime }$ using the minimum distance to Riemannian mean algorithm [26].5.Construct $S_{t}$$S_{b}$$S_{w}^{\left(c\right)}$L6.Repeat7.    Solve the generalized eigen-decomposition problem in (18) and select the k leading eigenvectors as the transformation $W^{*}$8.    Obtain the embedding features: ${\left\{z_{i}^{s}\right\}}_{i=1}^{Ns}$, ${\left\{z_{i}^{t}\right\}}_{i=1}^{Nt}$ by $z={\left(W^{*}\right)}^{T}\cdot K$.9.    Train a classifier f on ${\left\{z_{i}^{s},y_{i}\right\}}_{i=1}^{Ns}$ to update pseudo labels in target domain10.   Update L.11.Until convergence12.Obtain transformation matrix $W^{*}$ and target labels $Y_{t}$


The joint geometrical and statistical alignment (JGSA) [48] algorithm is a similar study to KMDA. JGSA mainly concentrates on finding two coupled projections that embed the source and target data into low-dimensional subspaces, where the domain shift is reduced while preserving the target domain properties and the discriminative information of source data, simultaneously. KMDA improves in two aspects. One is that, with the help of Riemannian alignment, KMDA transforms the source and target data into a common space, and hence it is reducible to solve an embedded subspace. Besides, the features in JGSA must be in the form of a flattened vector, while KMDA is characterized by the form of an SPD matrix.

(5) Converting Multichannel EEG Signals into 2D Frames

We assumed a set of EEG signals can be divided into M segments by a sliding window, denoted by $x_{i}\in R^{c\times m}$(i=1,2,…,M). 

In each segment, we calculated the power of each channel in the 8~30 Hz frequency band in sequence. *welch* (the built-in function package of MATLAB) was used first to calculate the power spectral density, followed by the *pwelch* function for the power spectrum and then the *bandpower* function to extract the power of the alpha and beta rhythm. As a result, we flattened a c×m EEG signal into a 1×c vector, with each element corresponding to the power value $x_{i}=[v_{1},v_{2},\ldots ,v_{c}]$. For the purpose of maintaining spatial information among multiple adjacent channels, we further converted the vector $x_{i}$ to a 2D frame according to the electrode distribution map. Figure 2a illustrates the schematic diagram for 2D EEG frames in the 22-electrode scenario, where the electrodes circled in bold are the selected ones. The constructed frame, $f_{i}$, is expressed as:

$$f_{i}=\left[\begin{array}{ccccc} \bar{v} & \bar{v} & v_{1} & \bar{v} & v\\ v_{2} & v_{3} & v_{4} & v_{5} & v_{6}\\ v_{8} & v_{9} & v_{10} & v_{11} & v_{12}\\ v_{14} & v_{15} & v_{16} & v_{17} & v_{18}\\ \bar{v} & \bar{v} & v_{20} & \bar{v} & \bar{v} \end{array}\right]$$

and $f_{i}$ must be a square matrix. We filled the central raw of the matrix $f_{i}$ with electrodes located in the central functional area (marked with C1, C2, …, Cn), partitioning the matrix into upper and lower parts, and each part was associated with the physical installation positions. Then, we completed the matrix with task-related electrodes, and the unused electrodes and the positions without electrodes were represented by the average power of all electrodes, $\bar{v}$. In this way, the chain-like EEG signals were converted to 2D frame sequences, $\left[f_{1},f_{2},\ldots ,f_{M}\right]$, and each frame, $f_{i}$, embodied the task-related power features and spatial information.

It is obvious that the size of a 2D frame is much smaller than that of an EEG signal, and is of great significance to improving the computation speed of Riemannian manifolds. Taking a 22-electrode setup as an example, the covariance matrix of an EEG trial is 22×22, whereas the size is 5×5 for a 2D frame. 

## 4. Experiments and Results

### 4.1. Dataset Description

BCI Competition IV Dataset IIa (Dataset IIa) consists of 9 subjects, ‘A01’, ‘A02’, …, ‘A09’. The 4-class cued motor imagery data were recorded by 22 EEG channels with a 250 Hz sampling rate. At t = 2 s, an arrow indicated that one of the four classes promoted the subject to perform the desired mental task until the cue disappeared at t = 6 s. Each subject recorded two sessions on different days, one for calibration, and the other for evaluation. Each session is comprised of 6 runs, and one run consists of 48 trials (12 trials per class), yielding 288 trails per session. In our experiment, we removed the trials containing an artifact label, marked with 1′ in the h.ArtifactSelection list.

BCI Competition III Dataset IVa (Dataset IVa) contains 2-class EEG signals recorded at 118 channels with a 1000 Hz sampling rate (down-sampled to 100 Hz in this paper) from 5 subjects, named as ‘AA’, ‘AY’, ‘AW’, ‘AL’, and ‘AV’. For each subject, a total of 280 cue-based trials are available. In each trial, a cue was indicated for 3.5 s, during which two MI tasks were performed: right hand and right foot. Then, the cue was intermitted by periods of random length, 1.75 to 2.25 s, in which the subject could relax.

BCI Competition III Dataset IIIa (Dataset IIIa) is a 4-class EEG dataset (left hand, right hand, foot, tongue) from 3 subjects (‘K3’, ‘K6’, ‘L1’), recorded by 60 channels, sampled at 250 Hz. The dataset consists of several runs, with 40 trials for each run. After the beginning of each trial, the subject rested in the first 2 s, then performed the indicated mental task from t = 3 s to t = 7 s. Each of the 4 cues appeared 10 times per run in a random order.

In our experiments, after the removal of EEG baseline drift, all datasets were filtered by a 6-order 8~30 Hz bandpass filter. The calibration and evaluation trials of Dataset IIa were extracted from the 2.5 to 4.5 s time interval recommended by the competition winner, and Dataset IVa and Dataset IIIa were extracted using a 3 s window after the cue onset at 0.5 s.

### 4.2. Experiment Design

We verified the merits of the proposed KMDA using three datasets, and compared it with the state-of-the-art domain adaptation algorithms. Table 1 presents the descriptions of the concerned methods. Except for MEKT, none of the other control algorithms were originally designed for EEG analysis, and we adapted them slightly to fit the experimental situation. 

Feature Extraction: MEKT maps the covariance matrices into the tangent space at the identity matrix, yielding a collection of corresponding tangent vectors, and the other algorithms concatenate the covariance matrices into flattened vectors. For the BCI Competition III Dataset IVa, for instance, the size of each trial was 118 × 300, and the size of its covariance matrix was 118 × 118, so the corresponding concatenated vector size was 1 × 13,924, and the vector in the tangent space was 1 × 7021. The high-dimensional vectors put forward a high demand for the size of the training set, otherwise, the model would be overfitted. Therefore, we proposed the method of converting multichannel EEG signals to 2D EEG frames to reduce the dimension. In the follow-up experiments, except for KMDA, all the control methods took the covariance matrices of 2D frames as input, so for the Dataset IVa, the dimension of the corresponding tangent vector was 1 × 66, and the dimension of the concatenated vector was 1 × 121. Both the covariance matrix of the 2D frames and the EEG signals are discussed in KMDA. For ease of differentiation, KMDA refers to the model with the covariance matrices of the 2D frames as input, while e-KMDA takes the covariance matrices of the EEG signals as input. The matrix dimensions before and after the conversion of the three datasets are shown in Table 2.

Hyper-parameter: Since the target data is assumed to be unlabeled, cross-validation is not applicable to the parameter determination. We set the parameters of (17) as α=μ=1 and β=0.1, the iterations T=15, and the dimension of subspace k=d, where d is the dimension of the SPD matrix. The hyper-parameters for the other algorithms were set according to the recommendations in the corresponding literature.

Classifier: The k-Nearest Neighbor Classifier (KNN) was used for all methods. To facilitate the calculation, we fixed k = 3 for all the experiments.

Data setting: All our experiments were carried out on the calibration data from three datasets. Since the feature distributions of ‘A08’, ‘AL’, and ‘K3’ were distinguishable, they were treated as the source data for the corresponding dataset, and the rest of the subjects were taken as the target. Therefore, we had 8 + 4 + 2 = 14 transfer scenarios.

Measurement: For the classification evaluation of the four tasks (Dataset IIa and Dataset IIIa), we opted for the Kappa coefficient recognized by BCI competition, while for binary classification (Dataset IVa), we used accuracy as the evaluation index.

### 4.3. Results

**Figure 3:**
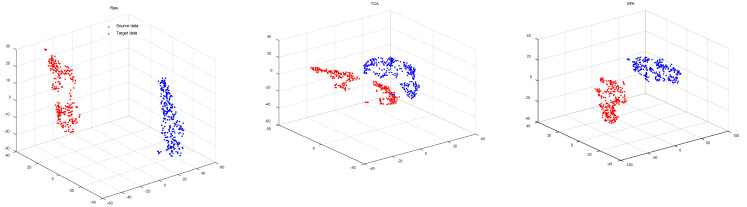

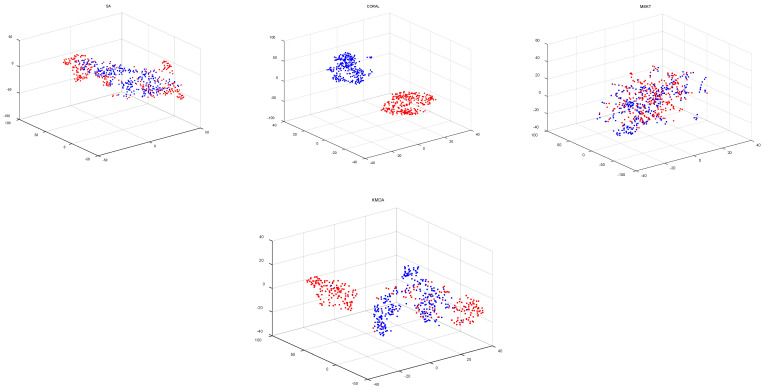
t-SNE visualization of the distributions with different unsupervised domain adaptation approaches.

Validation of Riemannian Alignment: Among the compared methods (as described in Table 1), except for JDA and JGSA, all the methods contained unsupervised distribution alignment of the source and target domains in Euclidean space or manifold space. Figure 3 visualizes the distributions after unsupervised alignment by the investigated methods in transferring subject ‘AL’ to subject ‘AA’ from DIVa by t-SNE [51]. The results indicated that the proposed RA not only aligns the marginal distributions of the source and target domain well, but also minimizes the distance between features of the two domains while preserving the characteristic of target distribution. Specifically, (a) to rectify the mismatch in distribution, TCA capitalizes on subspace learning [13], while GFK resorts to a shared space [50] to match the data distributions of different domains; however, both methods ignore the distribution characteristics of the target. (b) Both CORAL and SA align source data in the direction of the target domain. SA reconstructs the source data with the principal components of the target [49], and CORAL restructures the source data with all the eigenvectors of the target covariance matrix [47]. However, they fail to take into account the particularity of the covariance matrix as a feature, and the geometry of the SPD manifold. (c) The alignment approaches of MEKT and KMDA perform a parallel transport on the cone manifold of the SPD matrices to align the source with the target domain. However, MEKT whitens the covariance matrices of the source and target, resulting in an identical and uniform distribution [33], which completely destroys the characteristics of the target. By contrast, KMDA aligns the source covariance matrices with the target, yielding a set of covariance matrices formally similar to those of the target and consistent with the principal axis of the target, thus minimizing the domain shift while preserving the distribution characteristics of the target.

**Figure 4:**
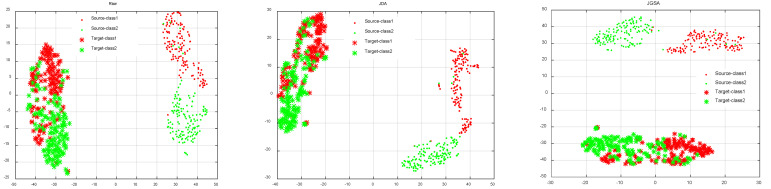

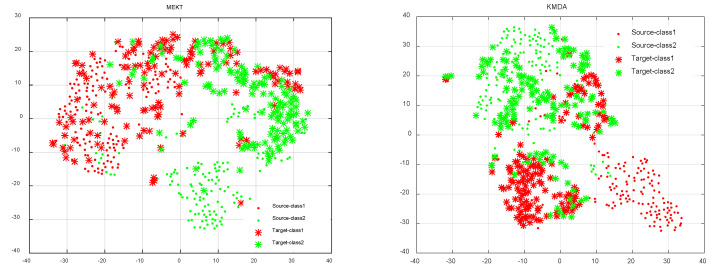
The visualization of transferring source data (subject AL with labels) to classify the unlabeled target data (subject AA) by JDA, JGSA, MEKT, and KMDA.

Validation of Subspace Learning: JDA, JGSA, MEKT, and KMDA aim to learn a discriminative subspace by leveraging labeled source data. Figure 4 depicts results of transferring subject ‘AL’ to subject ‘AA’ using the four domain adaption approaches. As shown in Figure 4, the raw source domain and target domain distribute differently, and their marginal distribution and conditional distribution are widely divergent. JDA and JGSA minimize the discrepancy of marginal and conditional distributions between the source and target, rather than the distance between features. MEKT and KMDA not only minimize the distribution divergence, but also make the features from the same class maximally close in the two domains. However, compared with MEKT, KMDA preserves more target distribution characteristics.

Classification accuracy: We evaluated KMDA and the other methods (list in Table 2) on different cross-domain scenarios. A baseline refers to the results of classifying the target data directly by a classifier trained on the source. Table 3 depicts the Kappa values of four metal tasks, and Table 4 shows the accuracies of Dataset IVa. We observed that the domain adaptation methods improved transfer performance to varying degrees. In general, KMDA achieved a better performance compared with other methods, the average Kappa values of KMDA were 0.56 and 0.75, and the average accuracy was 81.56%, 0.08, 0.05, and 5.28% higher than e-KMDA, respectively, which indicates that the 2D frame framework helps to improve performance. The results of a Wilcoxon signed rank test further confirmed the significant superiority of KMDA over other methods.

**Figure 5:**
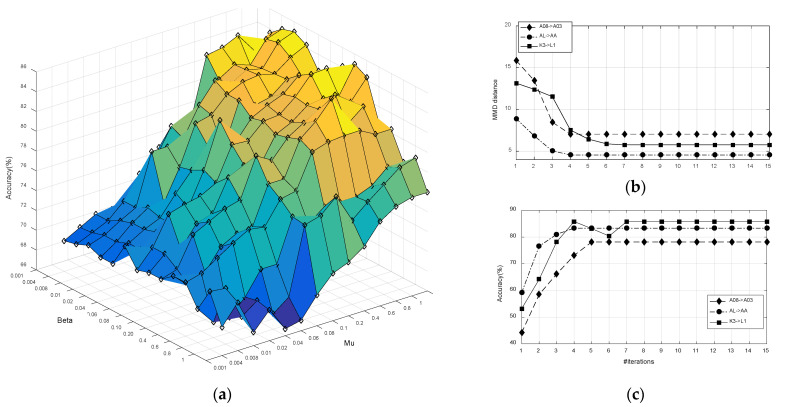
(**a**) Accuracies with different regularization parameters. (**b**) MMD distances and (**c**) accuracies on varying iterations.

Parameter Sensitivity: We analyzed the parameter sensitivity of KMDA in the scenario of ‘A08->A03’. The objective function of KMDA (17) contains three parameters, where α, μ, and β are trade-off parameters to balance the principal components of the target domain, the discrepancy of conditional probability distributions between the source and target, and the within- and between-class variance of the source, respectively. Since α only involves the target domain, and μ and β involve the source domain, the evaluation of α, μ, and β can be boiled down to the evaluation of μ and β under the condition of α = 1. Figure 5a demonstrates that the optimal values μ and β are not unique, and a large range of μ ($\mu \in [\begin{array}{cc} 0.4 & 1 \end{array}]$) and β ($\beta \in [\begin{array}{cc} 0.001 & 0.4 \end{array}]$) can be selected to obtain satisfactory performances. This is partly explained by the fact that, when the β exceeds 0.4, the model will overfit due to excessive attention to the discriminative information of the source.

**Figure 6:**
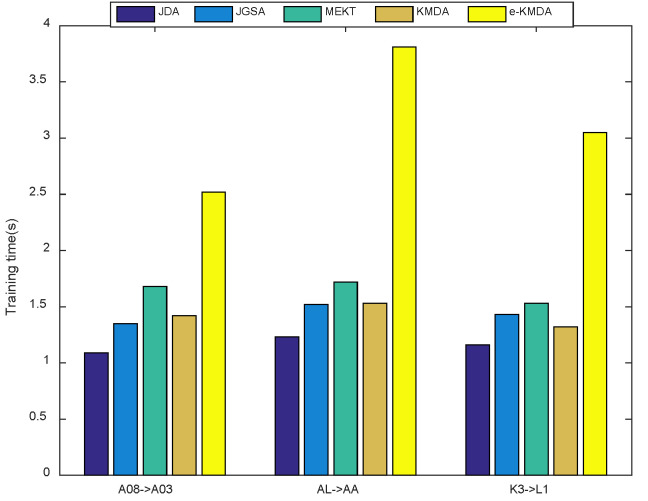
Training time of KMDA and other baseline methods.

Computation Complexity: We validated the convergence of KMDA, and checked the computation cost of the JDA, JGSA, MEKT, and KMDA/e-KMDA methods. Figure 5b,c demonstrate the results in ‘A08->A03’, ‘AL->AA’, and ‘K3->L1’ scenarios. As can be seen from Figure 5b,c in KMDA, the classification accuracy improved with the number of iterations, and the distribution distance gradually decreased and converged within 5 iterations. Figure 6 depicts the average running time of different algorithms above three scenarios, with the iterations. Although the proposed KMDA did not show an overwhelming advantage in terms of computation consumption, it was competitive under the trade-off of time and performance. Additionally, KMDA saved nearly half of the time compared to e-KMDA.

## 5. Discussion

### 5.1. Covariance Matrix as a Feature

The results in Table 3 and Table 4 not only prove the effectiveness of the KMDA (e-KMDA) method, but also imply that it is feasible to use the covariance matrix as a feature. Moreover, the results of KMDA (the covariance matrix of the 2D frame as a feature) are superior to those of e-KMDA (the covariance matrix of the EEG signal as a feature), which demonstrates that the framework for converting multichannel EEG signals to 2D frames improves the accuracy, accounting for the fact that it considers the electrode position and power spectrum characteristic of the signal. In order to further investigate the advantages of the 2D frame feature, we conducted a comparative experiment with the CSP-based variants. We compared KMDA (e-KMDA) with CCSP [25], SGRM [11], and CSP. Note that CSP is a supervised feature extraction method for binary data; for simplicity, only left- and right-hand mental data of Dataset IIa and Dataset IIIa were considered for this experiment. The source data were set according to the description of Section 4, with 20 trials per class with labels randomly selected from the target. Note that the KMDA (e-KMDA) prototype was designed for unlabeled target data. When considering the labels of the target data, we simply executed steps 7–9 of Algorithm 2 once. We opted to use RBF-based SVM (LibSVM Toolbox) as the classifier, whose parameters (-g gamma, -c cost) were determined by the built-in k-fold cross-validation function.

The results are depicted in Table 5. The classification accuracy was improved to varying degrees compared with the baseline CSP algorithm with the help of source data. The results of methods characterized by the SPD matrix were superior to the compared CSP variants with variance-based features. Specifically, the proposed KMDA method achieved the highest average accuracy of 78.54%, and e-KMDA came second with 75.79%. We conducted a Wilcoxon signed rank test on the accuracies to investigate the significance of the difference between KMDA and the other methods (*p* < 0.05). The results confirmed the significant superiority of KMDA (e-KMDA) over CSP in small target training sets.

### 5.2. Classification on Different Training Sets

Generally, the proposed KMDA is a transudative setting method, and so are the methods listed in Table 1. However, KMDA is also applicable to the classification of unseen test data. Herein, we explored the effectiveness of KMDA in an inductive setting, with varying numbers of labeled training samples from the target. We implemented this experiment on Dataset IVa and Dataset IIa, and the source data were set as stated in Section 4. For target setting, we considered only the left- vs. right-hand task of Dataset IIa, with calibration for training and evaluation data for testing. The Dataset IVa was divided in half, one for training and the other for testing. A given number of labeled samples were randomly selected from the training set, and the average accuracy of 5 repetitions was taken as the final output of the current subject.

SVM in tangent space (TSVM) [27,33,35] is a pervasive classifier for SPD matrices’ classification. To investigate the effectiveness of KMDA in the small-sample training set, we compared the results of TSVM with those of KMDA using SVM (LibSVM toolbox) as a classifier. Table 6 and Table 7 depict the accuracies of KMDA with different numbers of labeled training samples. It was observed that most results of KMDA outperformed those of TSVM, and the KMDA scores tended to be higher for the target subjects who performed well on TSVM, which demonstrated that KMDA could boost the classifier performance with knowledge from the source. However, we also found that KMDA domain adaptation was not always effective for all subjects in any scenario, and we expound the reason in the Limitations Section below.

### 5.3. Limitations

We further observed the results of Table 6 and Table 7 and found that, for subjects A03, A08, A09, and AL, when the training samples of the target were greater than 20 trials per class, the results of TSVM were higher than those of KMDA, i.e., the source data impaired the classification performance of the target, resulting in Negative Transfer (NT). The reason may be that for subjects who are good at motor imagery, their feature distributions are discriminative, while the source data becomes feature noise instead, hindering the generalization of the model. Due to the pervasive individual differences, the generalization from the source to the target is often limited by NT, unless the distributions of the source and target are close, and the tasks are similar [52]. To cope with NT, existing research can be summarized into four main categories: source data quality, target data quality, domain divergence, and integrated algorithms. For a more detailed survey on NT, please refer to [52]. Since the essence of the proposed KMDA is to learn cross-domain feature representation, we improved the performance of KMDA from two aspects: improving the quality of source data and reducing domain distribution discrepancy.

Source instance selection/weighting attempts to make the features of the source closer to those of the target by selecting similar instances or adjusting the weights. TrAdaBoost [53] is a typical instance-based boosting approach that increases the weight of the source instance if the corresponding instance is correctly classified, and vice versa. In addition, the similarity measures commonly used in the literature include Kellback–Leibler divergence [20], Cosine similarity [52], MMD distance [13,14,17], and domain transferability estimation [33]. In KMDA, we leveraged the pseudo-labels to calculate the conditional distribution distance of the embedded features. However, pseudo-labels and embedded features are two common unstable factors during transfer [52]. Therefore, it is of great significance to design a transfer model robust to feature noise (caused by embedded features) and class noise (induced by pseudo-labels) in the subspace learning process. For KMDA, we can introduce a sparse regularized term of the projection matrix into the objective function (15) to model the feature noise.

## 6. Conclusions

This paper proposed a kernel-based Riemannian manifold domain adaptation approach for motor imagery-based BCI classification. Compared to existing cross-subject EEG trial transfer works, KMDA (1) describes the EEG trials with their covariance matrices, (2) aligns the SPD matrices of sources and the target in the Riemannian manifold, and (3) exploits the Gaussian kernel based on the log-Euclidean metric to map the SPD matrices to a high-dimensional Reproducing Kernel Hilbert Space, then (4) performs domain adaptation by minimizing the probability distribution distance between the source and the target, while preserving the target’s distinct information and the discriminative information of sources. An optional descriptor of the EEG trial signal is presented to convert the chain-like EEG trial to a 2D framework, while preserving the spatial distribution. Extensive experiments on three motor imagery BCI datasets validated the effectiveness of KMDA in cross-subject adaptation. In brief, this paper presented a domain adaptation method that aims at transferring knowledge obtained from auxiliary EEG databases to the target subject, overcomes the subject dependence of the BCI system, and shortens the training time of the model. However, we note that not all source data produced positive effects. When the quality of target data was more discriminative than that of source data, the effectiveness of KMDA could not be guaranteed, resulting in negative migration. Under the framework of KMDA, we can further improve the effect of domain adaptation from two aspects: (1) by selecting similar instances or adjusting weights, the source features can be closer to the target features, so as to improve the quality of source data, and (2) by improving the generalization of classifiers trained on the source code, the discrepancies of domain distributions can be reduced.

## Figures and Tables

**Table 1:** Compared algorithms and parameters in the experiment.

Method	Descriptions	Para.
SA	A linear transformation on the principal components [49].	none
CORAL	Aligning the second-order statistics of the features [47].	none
GFK	The principal components of the source and the target are regarded as two points in the Grassmann manifold and a geodesic flow kernel (GFK) is obtained by integrating geodesics between the two points [50].	d<D/2
TCA	Minimizing the marginal probability distribution difference in RKHS [13].	none
JDA	Minimizing the joint distribution difference of marginal and conditional probability in RKHS [14].	λ=0.1
JGSA	Seeking two coupled projections that embed the source and target data into low-dimensional subspaces, where the domain shift is reduced while preserving the target domain properties and the discriminative information of source data simultaneously [48].	λ=1 μ=1 β=0.01
MEKT	Whitening the covariance matrices of source and target in Riemannian manifold, and learning two subspaces to reduce the domain divergences [33].	α=0.01 β=0.1 ρ=20
KMDA	Our algorithm.	

**Table 2:** Input space dimensions in different metrics.

No.	Raw EEG Trial			2D-Frame of Each Trial		
	CovD ^1^	TanV ^2^	ConV ^3^	CovD ^1^	TanV ^2^	ConV ^3^
Dataset IIa	22 × 22	1 × 253	1 × 484	5 × 5	1 × 15	1 × 25
Dataset IVa	118 × 118	1 × 7021	1 × 13,924	11 × 11	1 × 66	1 × 121
Dataset IIIa	60 × 60	1 × 1830	1 × 3600	9 × 9	1 × 45	1 × 81

^1^ CovD denotes the covariance matrix descriptor of the input signal. ^2^ TanV denotes the flattened vector of a covariance matrix in the tangent space. ^3^ ConV represents the concatenated vector of a matrix.

**Table 3:** Kappa statistics on cross-domain datasets. For each scenario, the highest value is marked in boldface. Note: *p*-values are derived by the Wilcoxon signed rank test between KMDA and each of the other methods.

**Dataset IIa**									
**Target**	**Baseline**	**SA**	**CORAL**	**TCA**	**JDA**	**JGSA**	**MEKT**	**KMDA ^1^**	**e-KMDA ^2^**
A08->A01	0.28	0.38	0.41	0.62	0.47	0.56	0.65	**0.68**	0.60
A08->A02	0.24	0.24	0.23	0.34	0.37	**0.43**	0.40	0.42	0.40
A08->A03	0.34	0.36	0.32	0.41	0.40	0.72	0.69	**0.74**	0.57
A08->A04	0.18	0.24	0.21	0.24	0.29	0.41	0.41	**0.44**	0.30
A08->A05	0.12	0.13	0.22	0.28	0.21	0.39	0.37	**0.40**	0.33
A08->A06	0.19	0.16	0.15	0.30	0.26	0.28	0.26	**0.31**	0.26
A08->A07	0.21	0.29	0.31	0.35	0.30	**0.64**	0.61	0.61	0.59
A08->A09	0.33	0.34	0.32	0.52	0.48	0.77	0.75	**0.78**	0.61
A03->A08	0.30	0.32	0.31	0.39	0.42	0.67	0.65	**0.69**	0.63
Average	0.24	0.27	0.28	0.38	0.36	0.54	0.53	**0.56**	0.48
*p*-value	*p* < **0.01**	*p* < **0.01**	*p* < **0.01**	*p* < **0.01**	*p* < **0.01**	0.1406	0.0823	-	*p* < **0.01**
**Dataset IIIa**									
**Target**	**Baseline**	**SA**	**CORAL**	**TCA**	**JDA**	**JGSA**	**MEKT**	**KMDA ^1^**	**e-KMDA ^2^**
K3->K6	0.26	0.42	0.4	0.44	0.54	0.65	0.64	**0.69**	0.63
K3->L1	0.29	0.41	0.44	0.51	0.63	0.75	0.68	**0.78**	0.72
K6->K3	0.22	0.52	0.48	0.65	0.68	**0.8**	0.73	0.78	0.75
Average	0.26	0.45	0.44	0.53	0.62	0.73	0.68	**0.75**	0.70

^1^ KMDA describes the covariance matrix of the 2D frame. ^2^ e-KMDA describes the covariance matrix with the EEG signal.

**Table 4:** Accuracy (%) on cross-domain datasets of Dataset IVa.

Target	Baseline	SA	CORAL	TCA	JDA	JGSA	MEKT	KMDA ^1^	e-KMDA ^2^
AL->AA	55.23	68.14	65.52	70.43	73.92	76.5	73.37	**78.57**	74.29
AL->AV	44.15	52.17	56.39	62.18	65.61	**71.69**	69.34	69.54	64.46
AL->AW	63.54	68.43	65.30	77.20	79.49	83.18	79.84	**84.32**	76.17
AL->AY	61.96	69.64	68.41	72.38	77.90	71.76	77.38	**79.70**	77.08
AY->AL	76.15	85.07	84.03	93.57	91.63	95.71	93.57	**95.71**	91.43
Average	60.21	68.69	67.93	75.15	73.71	79.77	78.7	**81.56**	76.28

^1^ KMDA describes the covariance matrix of the 2D frame. ^2^ e-KMDA describes the covariance matrix with the EEG signal.

**Table 5:** Classification accuracies achieved by CSP, CCSP, SGRM, KMDA, and e-KMDA with SVM being the classifier, respectively, on Dataset IIa and Dataset IIIa with 20 labeled trials per class. The highest accuracy of each subject is marked in bold. The *p*-values are derived by the Wilcoxon signed rank test between the results of KMDA (e-KMDA) and the other methods, respectively.

Subject	Left Hand vs. Right Hand				
	CSP	CCSP	SGRM	KMDA	e-KMDA
A01	65.28	71.11	73.85	**75.62**	74.13
A02	50.69	59.16	62.03	**63.56**	59.26
A03	83.33	84.71	**85.19**	80.09	79.38
A04	62.14	65.11	68.11	**72.13**	67.51
A05	57.64	62.70	66.38	**68.84**	64.35
A06	60.17	61.63	**69.61**	65.76	67.25
A07	67.36	79.40	84.06	**87.96**	81.64
A08	78.86	83.94	86.98	**89.69**	86.27
A09	**93.04**	81.36	87.76	90.66	89.22
K3	78.92	78.96	85.44	**92.08**	84.27
K6	66.37	70.07	**76.60**	**76.60**	74.47
L1	73.58	73.69	76.28	**79.53**	81.75
Ave.	69.78 ± 11.11	72.65 ± 8.34	76.86 ± 9.46	**78.54 ±** 9.18	75.79 ± 8.75
*p*-value	*p* < **0.01**	*p* < **0.01**	*p* < **0.05**	-	*p* < **0.01**
*p*-value	*p* < **0.01**	*p* < **0.01**	0.1722	*p* < **0.01**	-

**Table 6:** Classification accuracies achieved by KMDA and TSVM with varying numbers of labeled training samples from the target (per class), on Dataset IIa. The better results for KMDA are highlighted in bold, and the better results for TSVM are underlined.

		A01	A02	A03	A04	A05	A06	A07	A09	A08	Ave.
10 trials	TSVM	63.85	48.61	77.11	59.03	54.17	58.33	57.64	65.97	80.79	62.83
	KMDA	**68.76**	**54.32**	**80.75**	**62.47**	**61.01**	**63.19**	**63.41**	**72.06**	**83.89**	**67.76**
20 trials	TSVM	65.28	50.69	83.33	62.14	57.64	60.17	67.36	78.86	91.67	68.57
	KMDA	**75.62**	**63.56**	80.09	**72.13**	**68.84**	**65.76**	**87.96**	**89.69**	90.66	**77.15**
40 trials	TSVM	72.43	55.76	90.97	63.78	64.58	65.97	70.14	82.73	93.04	73.27
	KMDA	**76.19**	**69.8**	87.51	**70.41**	**68.4**	**69.96**	**87.33**	82.11	89.45	**77.91**
70 trials	TSVM	82.64	63.89	92.36	66.67	65.12	68.06	75.43	94.44	93.75	78.04
	KMDA	79.01	**72.52**	90.25	**70.25**	**68.55**	**71.02**	**88.29**	90.71	90.66	**80.14**

**Table 7:** Classification accuracies achieved by KMDA and TSVM with varying numbers of labeled training samples from the target (per class), on Dataset IVa. The better results for KMDA are highlighted in bold, and the better results for TSVM are underlined.

		AL->AA	AL->AV	AL->AW	AL->AY	AY->AL	Ave.
10 trials	TSVM	60.00	54.29	57.43	63.78	70.00	61.10
	KMDA	**65.21**	**63.78**	**67.09**	**68.21**	**74.02**	**67.66**
20 trials	TSVM	68.57	65.71	67.86	69.32	95.00	73.29
	KMDA	**75.02**	**69.03**	**71.73**	**70.02**	86.34	**74.43**
40 trials	TSVM	70.00	65.71	76.43	75.00	96.43	76.71
	KMDA	**78.34**	**70.32**	**78.32**	**76.34**	86.32	**77.93**
70 trials	TSVM	71.43	70.00	86.43	84.29	97.86	82.00
	KMDA	**80.11**	**73.03**	82.53	83.31	93.53	82.50

## Data Availability

Publicly available datasets were used in this research, that can be accessed via http://bbci.de/competition/iv/#download (18 April 2022) and http://bbci.de/competition/iii/#download (18 April 2022).

## References

[1] Lotte F., Bougrain L., Cichocki A., Clerc M., Congedo M., Rakotomamonjy A., Yger F. (2018). A Review of Classification Algorithms for EEG-based Brain–Computer Interfaces: A 10-year Update. J. Neural Eng..

[2] Zhang R., Li Y., Yan Y., Zhang H., Wu S., Yu T., Gu Z. (2018). Control of a Wheelchair in an Indoor Environment Based on a Brain–Computer Interface and Automated Navigation. IEEE Trans. Neural Syst. Rehabil. Eng..

[3] Yuriy M., Murat K., Erkan O., Yanar H. (2019). Developing a 3-to 6-state EEG-based brain–computer interface for a virtual robotic manipulator control. IEEE Trans. Biomed. Eng..

[4] Xu M., Han J., Wang Y., Jung T.-P., Ming D. (2020). Implementing Over 100 Command Codes for a High-Speed Hybrid Brain–Computer Interface Using Concurrent P300 and SSVEP Features. IEEE Trans. Biomed. Eng..

[5] Yu Y., Liu Y., Yin E., Jiang J., Zhou Z., Hu D. (2019). An Asynchronous Hybrid Spelling Approach Based on EEG–EOG Signals for Chinese Character Input. IEEE Trans. Neural Syst. Rehabil. Eng..

[6] Deepak K. (2022). Efficient Quadcopter Flight Control Using Hybrid SSVEP + P300 Visual Brain Computer Interface. Int. J. Hum. Comput. Interact..

[7] Anitha T., Shanthi N., Sathiyasheelan R., Emayavaramban G., Rajendran T. (2019). Brain–Computer Interface for Persons with Motor Disabilities—A Review. Open Biomed. Eng. J..

[8] Rodpongpun S., Janyalikit T., Ratanamahatana C.-A. (2020). Influential Factors of an Asynchronous BCI for Movement Intention Detection. Comput. Math. Methods Med..

[9] Chaisaen R., Autthasan P., Mingchinda N., Leelaarporn P., Kunaseth N., Tammaja S., Manoonpong P., Mukhopadhyay S.C., Wilaiprasitporn T. (2020). Decoding EEG Rhythms During Action Observation, Motor Imagery, and Execution for Standing and Sitting. IEEE Sens. J..

[10] Rong H.-J., Li C.J., Bao R.-J., Chen B.D. Incremental Adaptive EEG Classification of Motor Imagery-based BCI. Proceedings of the 2018 International Joint Conference on Neural Networks (IJCNN).

[11] Jiao Y., Zhang Y., Chen X., Yin E., Jin J., Wang X., Cichocki A. (2019). Sparse Group Representation Model for Motor Imagery EEG Classification. IEEE J. Biomed. Health Inform..

[12] Wu D., Xu Y., Lu B.-L. (2022). Transfer Learning for EEG-Based Brain–Computer Interfaces: A Review of Progress Made Since 2016. IEEE Trans. Cogn. Dev. Syst..

[13] Pan S.-J., Tsang I.-W., Kwok J.-T., Yang Q. (2011). Domain adaptation via transfer component analysis. IEEE Trans. Neural Netw..

[14] Long M., Wang J., Ding G., Sun J., Yu P.S. Transfer Feature Learning with Joint Distribution Adaptation. Proceedings of the 2013 IEEE International Conference on Computer Vision.

[15] Wang J., Chen Y., Hao S., Feng W., Shen Z. Balanced Distribution Adaptation for Transfer Learning. Proceedings of the 2017 IEEE International Conference on Data Mining (ICDM).

[16] Duan L., Tsang I.-W., Xu D. (2012). Domain transfer multiple kernel learning. IEEE Trans. Pattern Anal. Mach. Intell..

[17] Zhao K., Jiang H., Wu Z., Lu T. (2020). A novel transfer learning fault diagnosis method based on manifold embedded distribution alignment with a little labeled data. J. Intell. Manuf..

[18] Samek W., Vidaurre C., Müller K.-R., Kawanabe M. (2012). Stationary common spatial patterns for brain–computer interfacing. J. Neural Eng..

[19] Lu H., Eng H., Guan C., Plataniotis K.-N., Venetsanopoulos A.-N. (2010). Regularized Common Spatial Pattern with Aggregation for EEG Classification in Small-Sample Setting. IEEE Trans. Biomed. Eng..

[20] Samek W., Kawanabe M., Müller K. (2014). Divergence-Based Framework for Common Spatial Patterns Algorithms. IEEE Rev. Biomed. Eng..

[21] Cherlooa M.N., Amiri H.K., Daliri M.R. (2021). Ensemble Regularized Common Spatio-Spectral Pattern (Ensemble RCSSP) Model for Motor Imagery-based EEG Signal Classification. Comput. Biol. Med..

[22] Park S.-H., Lee D., Lee S.-G. (2018). Filter Bank Regularized Common Spatial Pattern Ensemble for Small Sample Motor Imagery Classification. IEEE Trans. Neural Syst. Rehabil. Eng..

[23] Zhang Y., Namand C.-S., Zhou G., Jin J., Wang X., Cichocki A. (2019). Temporally Constrained Sparse Group Spatial Patterns for Motor Imagery BCI. IEEE Trans. Cybern..

[24] Jin J., Xiao R., Daly I., Miao Y., Wang X., Cichocki A. (2021). Internal Feature Selection Method of CSP Based on L1-Norm and Dempster–Shafer Theory. IEEE Trans. Neural Netw. Learn. Syst..

[25] Lotte F., Guan C. (2011). Regularizing Common Spatial Patterns to Improve BCI Designs: Theory and Algorithms. IEEE Trans. Biomed. Eng..

[26] Barachant A., Bonnet S., Congedo M., Jutten C. (2012). Multiclass brain–computer interface classification by Riemannian geometry. IEEE Trans. Biomed. Eng..

[27] Barachant A., Bonnet S., Congedo M., Jutten C. Riemannian Geometry Applied to BCI Classification. Proceedings of the 9th International Conference on Latent Variable Analysis and Signal Separation.

[28] Kalunga E.-K., Chevallier S., Barthélemy Q., Djouani K., Monacelli E., Hamam Y. (2016). Online SSVEP-based BCI using Riemannian Geometry. Neurocomputing.

[29] Korczowski L., Congedo M., Jutten C. Single-Trial Classification of Multi-User P300-Based Brain–Computer Interface Using Riemannian Geometry. Proceedings of the 37th Annual International Conference of the IEEE Engineering in Medicine and Biology Society (EMBC).

[30] Yair O., Ben-Chen M., Talmon R. (2019). Parallel Transport on the Cone Manifold of SPD Matrices for Domain Adaptation. IEEE Trans. Signal Process..

[31] Rodrigues P.L.C., Jutten C., Congedo M. (2019). Riemannian Procrustes Analysis: Transfer Learning for Brain–Computer Interfaces. IEEE Trans. Biomed. Eng..

[32] Cai Y., She Q., Ji J., Ma Y., Zhang J., Zhang Y. (2022). Motor imagery EEG decoding using manifold embedded transfer learning. J. Neurosci. Methods.

[33] Zhang W., Wu D. (2020). Manifold embedded knowledge transfer for brain–computer interfaces. IEEE Trans. Neural Syst. Rehabil. Eng..

[34] Kumar S., Mamun K., Sharma A. (2017). CSP-TSM: Optimizing the performance of Riemannian tangent space mapping using common spatial pattern for MI-BCI. Comput. Biol. Med..

[35] Yger F., Berar M., Lotte F. (2017). Riemannian approaches in brain–computer interfaces: A review. IEEE Trans. Neural Syst. Rehabil. Eng..

[36] Uehara T., Sartori M., Tanaka T., Fiori S. (2017). Robust Averaging of Covariances for EEG Recordings Classification in Motor Imagery Brain–Computer Interfaces. Neural Comput..

[37] Wang R., Guo H., Davis L.-S., Dai Q. Covariance Discriminative Learning: A Natural and Efficient Approach to Image Set Classification. Proceedings of the 2012 IEEE Conference on Computer Vision and Pattern Recognition.

[38] Chen K.-X., Ren J.-Y., Wu X.-J., Kittler J. (2020). Covariance descriptors on a gaussian manifold and their application to image set classification. Pattern Recognit..

[39] Peng H., Lei C., Zheng S., Zhao C., Hu B. (2021). Automatic epileptic seizure detection via stein kernel-based sparse representation. Comput. Biol. Med..

[40] Harandi M., Hartley R., Lovell B., Sanderson C. (2014). Sparse Coding on Symmetric Positive Definite Manifolds Using Bregman Divergences. IEEE Trans. Neural Netw. Learn. Syst..

[41] Yger F., Sugiyama M. (2015). Supervised log-Euclidean metric learning for symmetric positive definite matrices. Comput. Sci..

[42] Jayasumana S., Hartley R., Salzmann M., Li H., Harandi M. (2015). Kernel methods on Riemannian manifolds with Gaussian RBF kernels. IEEE Trans. Pattern Anal. Mach. Intell..

[43] Arsigny V., Fillard P., Pennec X. (2011). Geometric Means in a Novel Vector Space Structure on Symmetric Positive-Definite Matrices. SIAM J. Matrix Anal. Appl..

[44] Horev I., Yger F., Sugiyama M. (2017). Geometry-Aware Principal Component Analysis for Symmetric Positive Definite Matrices. Mach. Learn..

[45] Pennec X., Fillard P., Ayache N. (2006). A Riemannian Framework for Tensor Computing. Int. J. Comput. Vis..

[46] Harandi M., Salzmann M., Hartley R. (2018). Dimensionality Reduction on SPD Manifolds: The Emergence of Geometry-Aware Methods. IEEE Trans. Pattern Anal. Mach. Intell..

[47] Sun B., Feng J., Saenko K. Return of Frustratingly Easy Domain Adaptation. Proceedings of the 30th AAAI Conference on Artificial Intelligence.

[48] Zhang J., Li W., Ogunbona P. (2017). Joint geometrical and statistical alignment for visual domain adaptation. arXiv.

[49] Fernando B., Habrard A., Sebban M., Tuytelaars T. Unsupervised Visual Domain Adaptation Using Subspace Alignment. Proceedings of the 2013 IEEE International Conference on Computer Vision.

[50] Gong B., Shi Y., Sha F., Grauman K. Geodesic Flow Kernel for Unsupervised Domain Adaptation. Proceedings of the 2012 IEEE Conference on Computer Vision and Pattern Recognition.

[51] Laurens V.D.M., Hinton G. (2008). Visualizing Data using t-SNE. J. Mach. Learn. Res..

[52] Zhang W., Deng L., Zhang L., Wu D. (2020). Overcoming Negative Transfer: A Survey. arXiv.

[53] Huang X., Rao Y., Xie H., Wong T.L., Wang F.L. Cross-Domain Sentiment Classification via Topic-Related TrAdaBoost. Proceedings of the 31st AAAI Conference on Artificial Intelligence.

